# Using Head-Mounted Ethanol Sensors to Monitor Olfactory Information and Determine Behavioral Changes Associated with Ethanol-Plume Contact during Mouse Odor-Guided Navigation

**DOI:** 10.1523/ENEURO.0285-20.2020

**Published:** 2021-01-15

**Authors:** Mohammad F. Tariq, Suzanne M. Lewis, Aliena Lowell, Sidney Moore, Jesse T. Miles, David J. Perkel, David H. Gire

**Affiliations:** 1 Graduate Program in Neuroscience, University of Washington, Seattle, Washington 98195; 2 Department of Psychology, University of Washington, Seattle, Washington 98195; 3 Departments of Biology and Otolaryngology, University of Washington, Seattle, Washington 98195; 4 University of Washington Institute for Neuroengineering, Seattle, Washington 98195

**Keywords:** behavior, foraging, navigation, olfaction, sensory

## Abstract

Olfaction guides navigation and decision-making in organisms from multiple animal phyla. Understanding how animals use olfactory cues to guide navigation is a complicated problem for two main reasons. First, the sensory cues used to guide animals to the source of an odor consist of volatile molecules, which form plumes. These plumes are governed by turbulent air currents, resulting in an intermittent and spatiotemporally varying olfactory signal. A second problem is that the technologies for chemical quantification are cumbersome and cannot be used to detect what the freely moving animal senses in real time. Understanding how the olfactory system guides this behavior requires knowing the sensory cues and the accompanying brain signals during navigation. Here, we present a method for real-time monitoring of olfactory information using low-cost, lightweight sensors that robustly detect common solvent molecules, like alcohols, and can be easily mounted on the heads of freely behaving mice engaged in odor-guided navigation. To establish the accuracy and temporal response properties of these sensors we compared their responses with those of a photoionization detector (PID) to precisely controlled ethanol stimuli. Ethanol-sensor recordings, deconvolved using a difference-of-exponentials kernel, showed robust correlations with the PID signal at behaviorally relevant time, frequency, and spatial scales. Additionally, calcium imaging of odor responses from the olfactory bulbs (OBs) of awake, head-fixed mice showed strong correlations with ethanol plume contacts detected by these sensors. Finally, freely behaving mice engaged in odor-guided navigation showed robust behavioral changes such as speed reduction that corresponded to ethanol plume contacts.

## Significance Statement

Animals are remarkable at locating resources, essential to survival, from long distances using olfactory information. Understanding the neurophysiological mechanisms and the behavioral strategies for accomplishing this goal has been challenging because of the intermittent nature of the olfactory information. Here, we present a method to record real-time olfactory information using low cost, lightweight sensors that can be mounted on the head of freely behaving rodents. This technology can therefore allow us to better correlate the spatiotemporally varying intermittent olfactory information with behavioral changes and neurophysiological recordings.

## Introduction

Animals from different phyla within the kingdom use olfactory cues to guide navigation and decision-making to find resources essential to survival. This task of locating resources using olfaction is a complex problem because of the nature of the olfactory stimulus. Odor molecules emanating from a source spread because of turbulent air currents (Celani and Seminara, 2005; Celani et al., 2014). These dynamically varying currents result in stochastic mixing of the odor molecules with the fluid molecules. The net result of these turbulent forces is a dynamically varying spread of odor molecules in time and space as odor plumes. Hence, time-averaged concentrations of the odorant in space are a poor measure of the olfactory information that a freely moving searcher, using odors to find the source, will experience (Baker et al., 2018). Measuring this odor input in real time, as sensed by the searcher, is essential to understanding how the nervous system processes olfactory information during complex free behavior such as plume tracking.

Current technologies for chemical quantification are not feasible for mobile operations. Photoionization detection (PID) is a technology that is routinely used by olfactory researchers (Riffell et al., 2008); but the sensor in this case is also relatively large and expensive. A technology used to study odor-guided navigation by insects is electroantennography (EAG; Justus et al., 2005). EAG sensors are small, lightweight and highly sensitive to volatile compounds. However, EAG signals are also highly sensitive to changes in air pressure and temperature, and often degrade over the time scale of hours, which precludes the use of the same EAG sensor on multiple animals. We thus employed a method for using lightweight and low-cost metal oxide gas sensors that are used in environmental chemical detection systems (Wang et al., 2010) as a feasible method for relatively rapid mobile monitoring of the odor environment.

One limitation of using the metal oxide sensors is the long decay time of the sensor recordings in response to transient activation with the chemical stimulus. Here, we first conducted paired alcohol-sensor and PID recordings to pulsed and naturally fluctuating ethanol stimuli and developed a method to deconvolve the sensor recordings to improve the response time and increase the match with PID recordings. This deconvolution method is similar to approaches that have been successfully used in robotics contexts (Martinez et al., 2019), and produces similar results. Furthermore, we present results correlating the deconvolved alcohol-sensor signals to calcium imaging responses in head-fixed mice. Additionally, results from freely navigating mice engaged in localizing an ethanol odor source show speed reductions on ethanol plume contacts detected by the head-mounted alcohol sensors. This method can serve as a cost-effective way to correlate real-time olfactory information with behavior and physiological responses during olfactory navigation tasks.

## Materials and Methods

The designs for the 3D-printed parts and software used to analyze the alcohol sensor recordings will be publicly available at the author’s website on publication.

### Paired PID and ethanol-sensor recordings

Figaro TGS 2620 Organic Solvent Vapor Sensor [powered by a 5V DC voltage from an Arduino (Adafruit)] was used to monitor the relative concentration of ethanol vapors in the air. To prevent extended contact of odor-laced air with the sensor, the head cap of the sensor was removed. Paired PID and alcohol sensor recordings were then conducted ∼10–15 mm downstream of an odor port releasing ethanol vapors from a tube controlled by a valve (Clippard EV-2-12). The PID and sensor recordings were digitized by NI USB-6009 DAQ (National Instruments) at a sampling frequency of 500 Hz. The data acquisition and valve control were conducted through custom scripts written in LabView (National Instruments). For recordings in a dynamic plume, paired recordings were conducted downwind of an ethanol port in a custom-designed arena (see Results section) at multiple locations. Data from locations near the port, near the middle of the arena, and farthest downwind were pooled.

### Deconvolution of the ethanol-sensor signal

The raw alcohol sensor recordings were low-pass filtered using a digital filter in MATLAB (MathWorks; cutoff frequency, 40 Hz). The frequency content in the filtered signal was then obtained by the *fft* routine. A difference-of-two-exponentials kernel (Monroy et al., 2012) was then designed (see Results) using the equation:

$$f\left(t\right)=\exp\left(-\frac{t}{\tau_{Decay}}\right)-\text{exp}\left(-\frac{t}{\tau_{Rise}}\right).$$

The frequency content of the kernel, obtained by the *fft* routine, was then used to divide the filtered signal. The resulting spectrum was then converted back into the time domain by taking the *ifft* of the signal. The difference between the PID recording and the deconvolved signal was calculated by normalizing both signals to their maximum values. This allowed for the comparison of the waveform of the two signals with no particular emphasis on the amplitude of the responses. Values for $\tau_{Rise}$ of 2 ms and $\tau_{Decay}$ of 0.5 s minimized the least-squares error between the deconvolved sensor and the PID signals.

### Animal care

All animal procedures were performed in accordance with the University of Washington institutional animal care committee’s regulations. All mice strains were obtained from The Jackson Laboratory and maintained in local colonies with *ad libitum* food supply. After surgical procedures, each animal was singly housed. The colony was maintained on reverse 12/12 h light/dark cycle (7 A.M. lights off; 7 P.M. lights on) with behavioral experiments performed during the dark cycle [zeitgeber time (ZT)12–ZT23].

### Calcium signals from the olfactory bulbs of awake animals

For calcium imaging of active glomeruli in response to alcohol plumes, an imaging window was placed over the dorsal main olfactory bulb (OB) of Thy1-GCaMP6f-GP5.11 (Dana et al., 2014) mice using methods, described previously (Batista-Brito et al., 2017). Following recovery from the surgery, imaging sessions were conducted while each mouse was head-fixed under a widefield microscope within a wind tunnel during alcohol plume presentations. In the wind tunnel the odor was carried downwind from an odor port located 13.5 cm from the nose of the head-fixed animal. To record ethanol signals, an alcohol sensor was placed within 4 mm of the right nostril. The calcium signals were then spatially segmented using constrained nonnegative matrix factorization (CNMF) (Pnevmatikakis et al., 2016), and the alcohol sensor recordings deconvolved (see Materials and Methods). In the wind tunnel odors were transported as turbulent plumes, making the time of arrival variable from trial to trial. The calcium responses from each glomerulus were therefore aligned with respect to the first peak recorded by the ethanol sensor for each trial to study the ethanol-evoked responses within the projection neurons of the OB.

### Behavioral apparatus

The arena to study plume tracking in freely moving animals was constructed as reported previously (Jackson et al., 2020). Briefly, the arena [2 m (l), 0.9 m (w), 0.9 m (h)] was constructed using aluminum railings and closed off using clear acrylic along the length. Furthermore, a clear acrylic sheet was placed on top to close off the air supply. The floor was a sheet of opaque white acrylic. The two smaller ends were covered by a thin film of mesh to allow air currents to pass in a directional manner. Furthermore, an exhaust fan spanning the width of one half of one end of the arena created a directional air movement pattern. The nesting area was placed outside the arena and constructed from clear acrylic, with a small passage into the arena gated by a motor under LabView control. A custom designed mechanism, under Arduino control, was used to position the odor port (5- to 10-mm distance from the arena floor) at any *x-y* location within the arena. The odor stimulus consisted of passing air, controlled by valves (Clippard EV-2-12), from a tube filled with pure ethanol, resulting in ethanol vapor introduced via a clear plastic tube through the odor port into the arena. Colocalized with the odor port was the port to deliver reward (water droplets), which was also controlled with valves. An overhead camera (Basler acA640-90uc; frame rate, 90 fps) was used to record the behavior and the position of the odor and reward ports within the arena.

### Simultaneous behavioral and olfactory information tracking from freely moving mice

Five WT littermate mice (five months old at implantation; two males/three females; strain no. 024105 The Jackson Laboratory; C57BL/6J background) were used as subjects. Each mouse underwent aseptic surgery, with isoflurane as anesthetic, to affix a 3D-printed sensor mount, which housed four magnets (K&J Magnetics D101-N52), to the skull. Separately, the sensor was affixed to a second 3D-printed part, which contained four magnets in opposite polarity to the head-mounted part. This part could nest inside the head-mounted part. This configuration allowed rapid placement and removal of the sensor on the animal’s head. After surgery, each mouse was housed singly.

Following a rest period (more than two weeks) to recover from surgery, mice were water deprived and trained to associate water reward with the ethanol odor. This training was achieved by placing a weigh boat, containing water reward with an ethanol-soaked pad taped to the bottom, in the home cage for at least 15 min. The training lasted for more than one week and was conducted during the dark phase (ZT12–ZT23). Each mouse was then maintained at >85% of the predeprivation body weight. After one week of association, animals were acclimatized to handling and restraining by the experimenters during 5- to 10-min sessions for each mouse. Each mouse then underwent further sessions while wearing a head-mounted sensor in its respective cage. Following the association and acclimatization with the sensor, each mouse was then introduced into the arena for exploration in 15-min sessions over the course of 3 days. The association was conducted in a different room than the room housing the arena.

To remove visual and auditory cues during the odor-source localization task within the behavioral arena, all the trials were conducted under infrared illumination, and with white noise played continuously on the speakers. Each trial started by first placing the odor port at one of the six chosen locations within the arena and turning on the odor stimulus. The gate from the nesting area was then opened and the mouse allowed to enter the arena. Once the mouse entered the arena, the gate to the housing cage was closed and the mouse was allowed to navigate to the location of the odor port to receive the reward. For the initial few trials, water droplets were dropped onto the floor of the arena until the animal improved in localizing the odor port. The order of the location of the odor port and the animal testing order was randomized each day.

Once the animal navigated to the odor port and received the water reward, a 1-kHz tone played for 1 s, signifying the end of trial and the gate to the nesting area opened. During the period from the end of the trial and the return of the animal to the nesting arena, the odor stimulus was turned off. For trials where the mouse took longer than ∼5 min to return to the arena, the mouse was gently coaxed back into the nesting area by an experimenter. Each mouse completed 4–12 trials each day. Once each animal was proficient in localizing the odor port, assessed qualitatively, trials with the head-mounted sensors were conducted in the behavioral arena. The data presented here are only from the trials when the sensor was mounted during the behavior.

### Analysis of the real-time olfactory information and behavior from freely behaving mice

All analysis software was written in MATLAB 2019a. The videos were analyzed by foreground and background separation using Dynamic Mode Decomposition (Grosek and Kutz, 2014). The body was discernible because of the dark fur against white background, while the head position was obtained using an LED mounted on the sensor body during the behavior. The resulting pixel cloud was then used to estimate the center of mass, allowing determination of the body and head positions. These position coordinates were further low-pass filtered to remove jitter. Speed was calculated as the distance traveled between each successive frame. The alcohol sensor recording was deconvolved as outlined above, but the $\tau_{Rise}$ constant was adjusted to 20 ms and the $\tau_{Decay}$ constant was adjusted to 2.0 s to minimize spurious detection of small signals because of head movement. The trajectories and sensor recordings for each trial were analyzed only between when the animal entered the arena and when it received the reward. User-defined thresholds for event crossings, detected by Schmitt Trigger (MathWorks File Exchange), were then set for the deconvolved signal from each trial. The deconvolved signal was further normalized by calculating the fractional deviation from the average baseline signal (mean of the signal during the initial 1 s on entry of the animal into the arena) as dx/x. Only event crossings separated by greater than 5 s were considered as separate plume encounters. Furthermore, the animal had to be further than 10 pixels from the odor source at the time of the event crossing. A total of 177 contacts from 88 trials passed these criteria. The body and head speeds, and the normalized deconvolved alcohol signal was then obtained in a 4-s window surrounding plume contacts, and these signals were averaged to obtain the mean time course. As control, equal time-sized portions of trajectories that did not contain plume contact were randomly selected.

## Results

### Difference of two exponentials kernel to deconvolve the raw alcohol sensor responses

**Figure 1.**
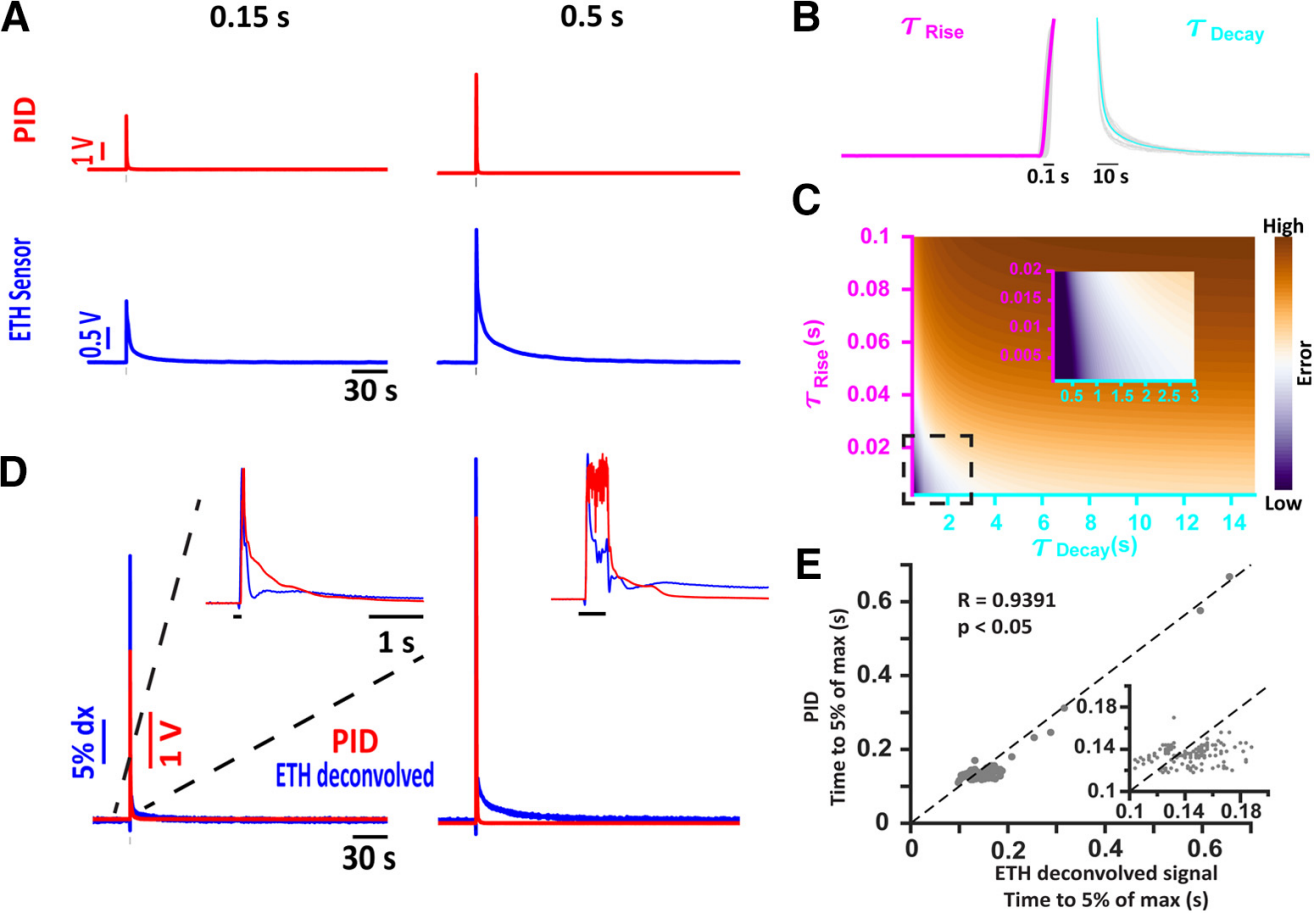
Ethanol-sensor response can be deconvolved using a difference of two exponential kernel. ***A***, Representative traces of the PID (red) and the ethanol-sensor (blue) response to brief ethanol presentations. The duration of the ethanol pulse is indicated at the top of each column. ***B***, Ethanol-sensor responses from individual presentations (gray) were averaged to estimate the rise time (τ_Rise_) to be around 60 ms (magenta) and the decay time (τ_Decay_) to be around 3.5 s (cyan). ***C***, Optimization of the deconvolution kernel by minimizing the mean error between the deconvolved ethanol responses and the PID responses (see Materials and Methods) using a family of kernels with varying τ_Rise_ and τ_Decay_ values. ***D***, The deconvolved ethanol signal (blue) from the raw recordings of the ethanol sensor shown in ***A*** compared with the PID recordings. The inset shows the zoomed-in view of the normalized deconvolved ethanol response and the PID response to compare the waveforms of the PID response and the deconvolved ethanol signal. The black bar in the inset shows the duration of ethanol presentation. ***E***, A linear relation exists between the threshold time (time from the valve opening to 5% of the max) between the deconvolved ethanol signals and the PID responses. Inset shows the zoomed-in view of the cluster of points in the ranges shown.

We first tested the feasibility of the ethanol sensor to record behaviorally relevant odor stimuli by conducting paired PID and ethanol-sensor recordings in response to brief ethanol stimuli (Fig. 1*A*). As can be seen, the ethanol-sensor recordings decay more slowly than the PID responses. To improve our understanding of the rise time and decay time of the ethanol-sensor responses, the individual recordings were averaged to determine the mean time rise and tau decay. The mean rise and decay signals (Fig. 1*B*) were then exponentially fit to obtain the $\tau_{Rise}$ (0.06 s) and $\tau_{Decay}$ (3.5 s). Modeling the ethanol response as a difference of two exponentials (Monroy et al., 2012); we deconvolved individual ethanol-sensor recordings using a family of kernels with a range of tau rise and tau decay values. These deconvolved signals were then compared with the PID recordings, taken as the ground truth. The dependence of the mean error between the deconvolved sensor recordings and the PID recordings as a function of tau rise and tau decay values of the kernel is presented in Figure 1*C*. Based on this information, we set the value of $\tau_{Rise}$ for the kernel to be 2 ms and that of $\tau_{Decay}$ to be 0.5 s. Using this kernel, the raw ethanol recordings presented in Figure 1*A* were deconvolved and are shown in Figure 1*D*. For comparison, the PID response is shown in red. The inset shows the normalized PID and deconvolved response to better compare the waveform of the two sensors. To test whether a major shift occurred because of the deconvolution procedure, we compared the threshold-crossing times (time to 5% of the max from the valve opening) of the deconvolved signal and the PID responses (Fig. 1*E*) for individual ethanol presentations. A linear relation (*r* = 0.9391, *p* < 0.001) exists between the threshold-crossing time of the deconvolved signal and the PID responses. This linear relationship decreased if only data presented in the inset of Figure 1*E* are used to compute the correlations (*r* = 0.1991, *p* = 0.0139). This decrease in correlation could be because of the different dynamic ranges, and active versus passive nature of the PID and the alcohol sensor (see Discussion).

**Figure 2.**
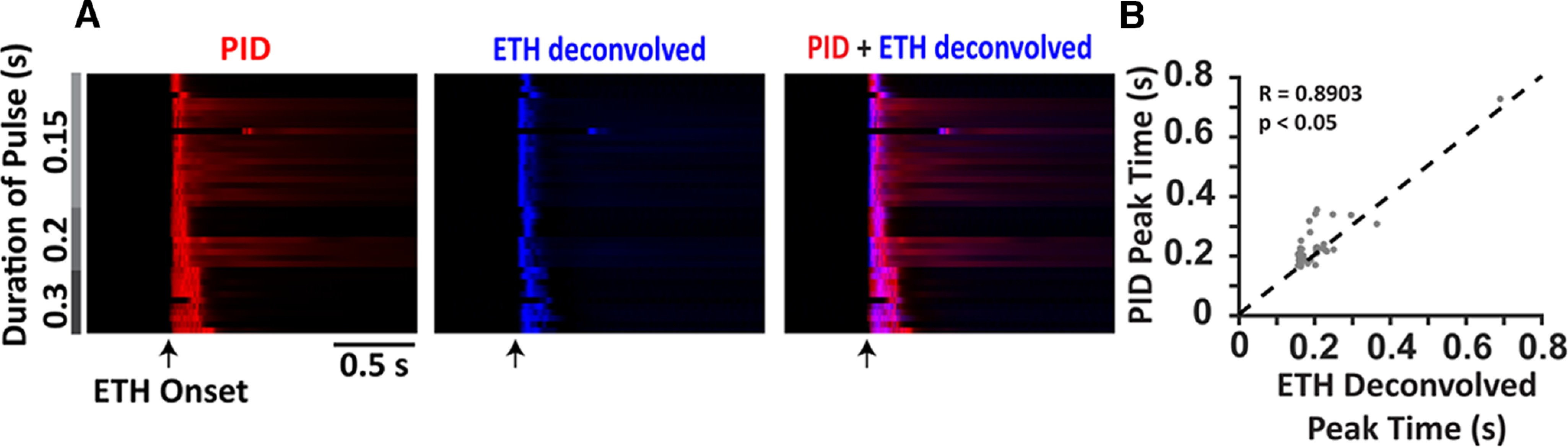
Peak times of the deconvolved ethanol signals coincide with the peak times of the PID responses for brief pulses of ethanol. ***A***, Summary data of PID responses (red; left) and the deconvolved ethanol signals (blue; center) to brief durations of ethanol pulses (shown in gray) presented as a heatmap where each row is a single trial aligned with respect to the valve opening (arrow). Overlaying the deconvolved ethanol signals over the PID responses (right) shows coincidence of the peaks from the two signals (magenta). ***B***, Linear relation between the peak times of the PID responses and the peak times of the deconvolved ethanol signals.

The summary data from brief pulses (0.3 s and less) are presented in Figure 2*A* as a heatmap where each row represents a single trial aligned with respect to the valve opening. The PID responses are presented in red, while the deconvolved ethanol-sensor signals are presented in blue. Overlaying the two responses we can see coincident activity in magenta (right). Furthermore, the peak times (time of the maximum amplitude response with respect to the valve opening) of the deconvolved signal and the PID response (Fig. 2*B*) show a linear relationship (*r* = 0.8903, *p* < 0.001) indicating the coincident activity of the two sensors. Deconvolution of the ethanol-sensor signal thus provides an adequate approximation of the real-time odor concentration.

### Frequency characteristics of the ethanol sensor

**Figure 3.**
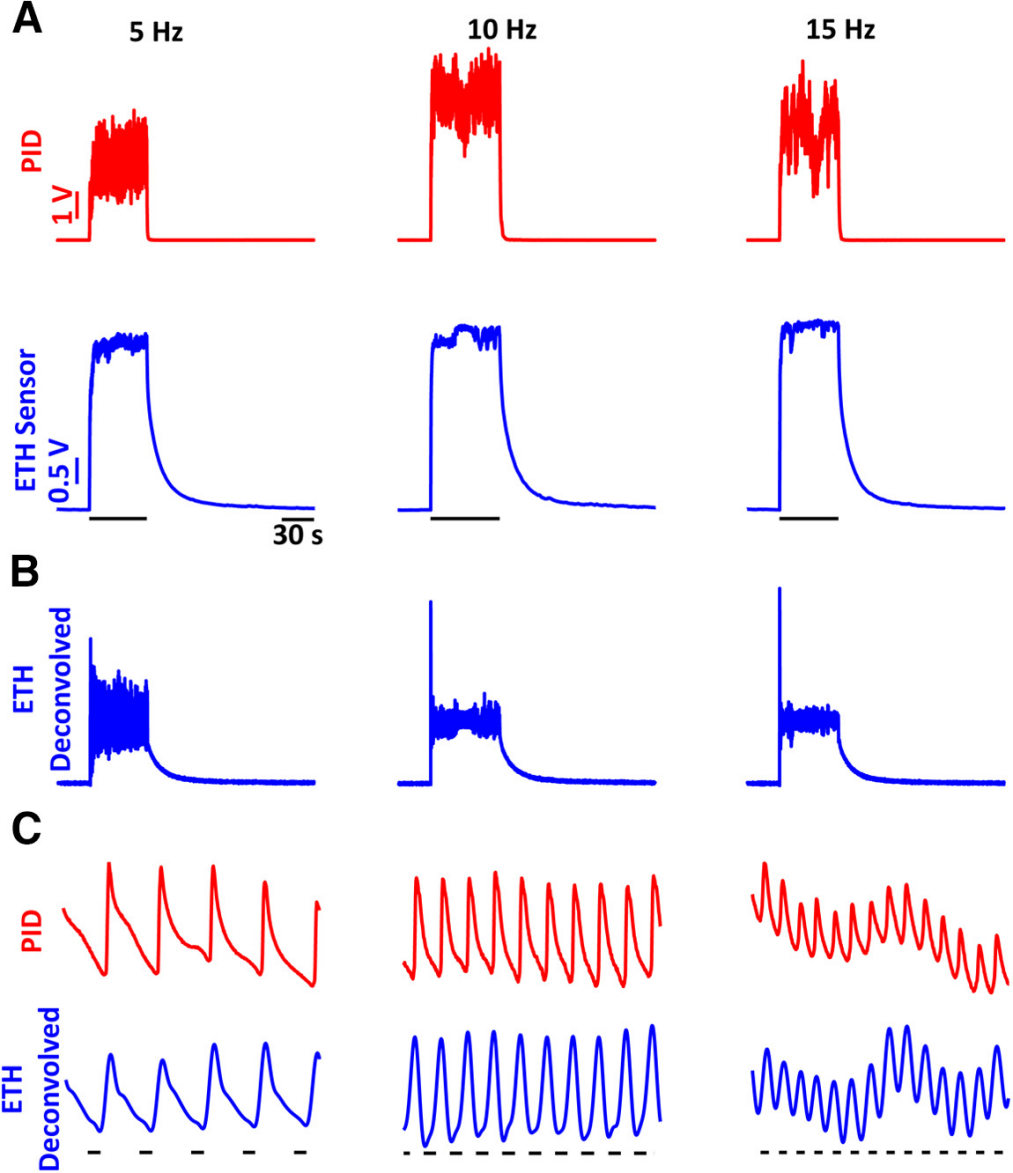
Frequency responses of the ethanol sensor show that the sensor can resolve frequencies of ethanol fluctuations up to 15 Hz. ***A***, PID (red) and ethanol-sensor (blue) responses to single trials of ethanol fluctuations of 5 Hz (left), 10 Hz (center), and 15 Hz (right) frequencies. ***B***, Deconvolved signals of the raw ethanol-sensor recordings presented in ***A***. ***C***, A 1-s zoomed-in view of the PID responses (red) and deconvolved ethanol signal (blue) showing near identical responses in the deconvolved signals and the PID recordings. The black bars underneath the traces indicate the times when the valve was open.

We then tested the range of frequencies at which the ethanol sensor can detect signals by conducting paired PID and alcohol-sensor recordings to ethanol pulse stimuli of 5-, 10-, and 15-Hz frequencies. Figure 3*A* shows the raw ethanol sensor and PID recordings for single trials of the frequencies tested. The raw sensor recordings were then deconvolved using the same kernel as used for the single pulses (see above). The deconvolved signal is shown in Figure 3*B*. To better compare the deconvolved signal and the PID recordings, 1-s expanded displays of the signals are presented in Figure 3*C* showing nearly identical responses to the ethanol fluctuations.

**Figure 4.**
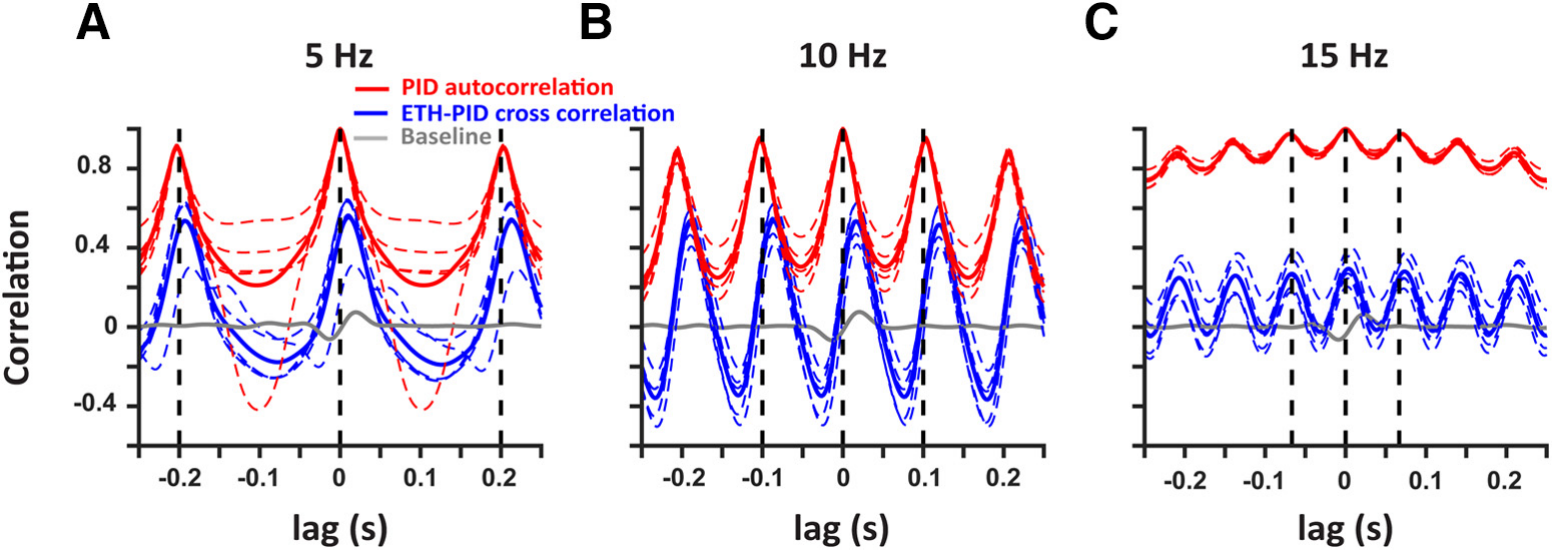
Deconvolved ethanol signals are correlated with the PID signals at multiple frequencies. Cross-correlation of the deconvolved ethanol signal with the PID signal during stimulation (blue) at 5 Hz (***A***), 10 Hz (***B***), and 15 Hz (***C***) shows robust correlation as compared with baseline (gray). In addition, autocorrelation of the PID signals within each trial are also presented (red). The colored dashed traces represent individual trials and solid traces represent the mean. The black vertical dashed lines indicate the period for each frequency.

To better compare the frequency responses across multiple trials (*n* = 5 for each frequency), we conducted a cross-correlation analysis of the ETH deconvolved signals with respect to the PID signals during the stimulation (blue) and baseline windows (gray; Fig. 4). In addition, the auto-correlation of the PID signals within the trials are also presented (red). The mean cross-correlation (solid lines) and individual trials (dashed traces) show strong periodicity with peaks at the period of the respective frequencies (dashed black lines). There is a consistent small difference in the peak times of the mean cross-correlation with respect to the period across all the frequencies which can be explained by the active suction of the PID. Hence, alcohol sensors can faithfully record ethanol fluctuations up to at least 15 Hz.

### Ethanol-sensor and PID responses at varying distances from a plume source

**Figure 5.**
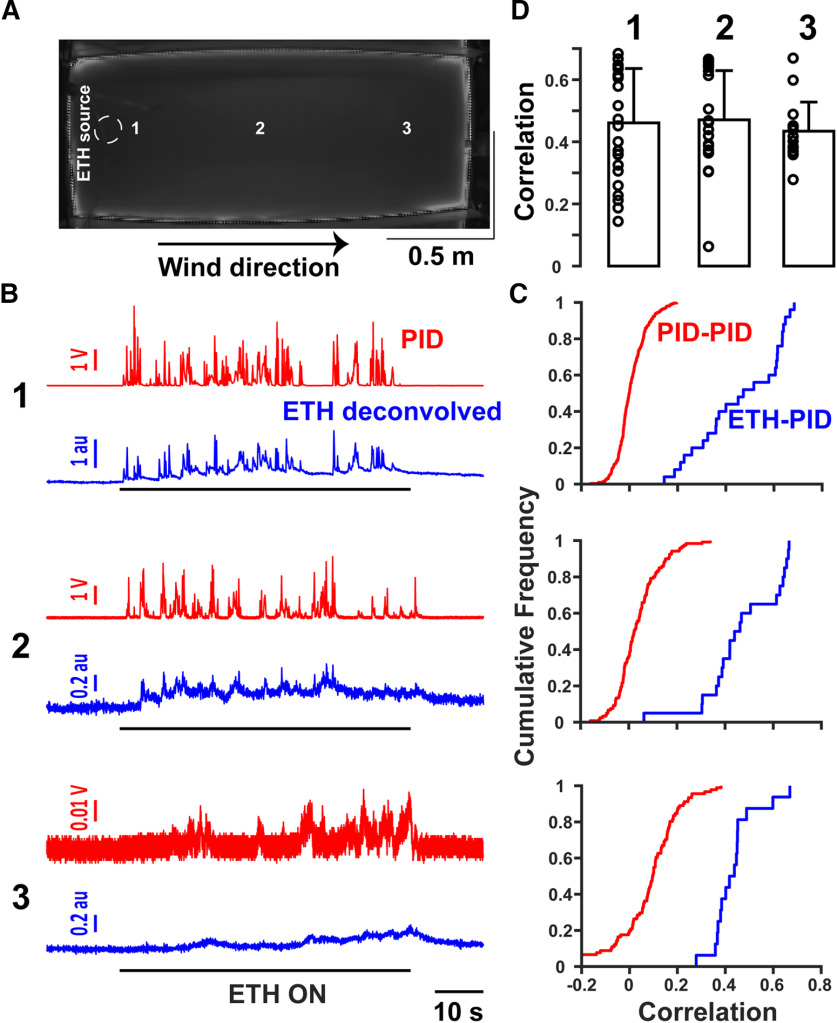
Deconvolved ethanol signals are correlated with the PID responses in turbulent airflow. ***A***, Overhead image of a custom-designed arena [2 m (l), 0.9 m (w), 0.9 m (h)] to create dynamic odor plumes. The dashed circle represents the location of the source of ethanol port while the arrow points out the wind direction. Also indicated are the approximate locations near the port (1), near the middle of the arena (2), and farthest downwind (3) where paired PID and ethanol-sensor recordings were conducted. ***B***, Representative traces of the PID (red) and deconvolved ethanol (blue) signals at the locations indicated in ***A***. The black bars represent the ethanol stimuli. Notice the changing scales for the amplitude at farther distances from the ethanol port. ***C***, Cumulative distributions of PID correlations across pair-wise combinations of different trials (red) versus correlations between the PID and the ethanol signals within a trial (blue) for the different locations. ***D***, Mean ± standard deviation (SD) correlations (bars) at locations near port (1), near the middle of the arena (2), and farthest downwind (3) from the ethanol port. Circles are the correlations of individual trials.

To test how distance from the ethanol source and turbulent mixing affected the alcohol sensor reading, we conducted paired PID and alcohol-sensor recordings in a custom-made arena designed to create dynamic odor plumes (Fig. 5*A*). The recordings were conducted near the source (1), near the middle of the arena (2), and farther downwind (3). Representative traces from the three locations are shown in Figure 5*B*. Increasing distance results in a decrease of the amplitude of both the PID and sensor readings. We calculated the correlation of the PID signals from all pair-wise combinations of distinct trials. The distribution of correlation values was near zero, suggesting that on different trials the plumes were highly distinct. In contrast, when we calculated the correlation of PID and deconvolved ethanol signals from the same trial, they were strongly correlated (Fig. 5*C*). The mean correlations [mean ± SD: 0.460 ± 0.175 (1), 0.4708 ± 0.1584 (2), 0.4343 ± 0.0937 (3)] between the PID and the deconvolved alcohol signal for the ethanol duration window are presented in Figure 5*D*. These correlation values indicate that the ethanol sensors can capture fluctuating signals across a wide range of ethanol concentrations and locations within the arena.

### Calcium imaging response onsets coincide with plume contacts detected by the ethanol sensor

**Figure 6.**
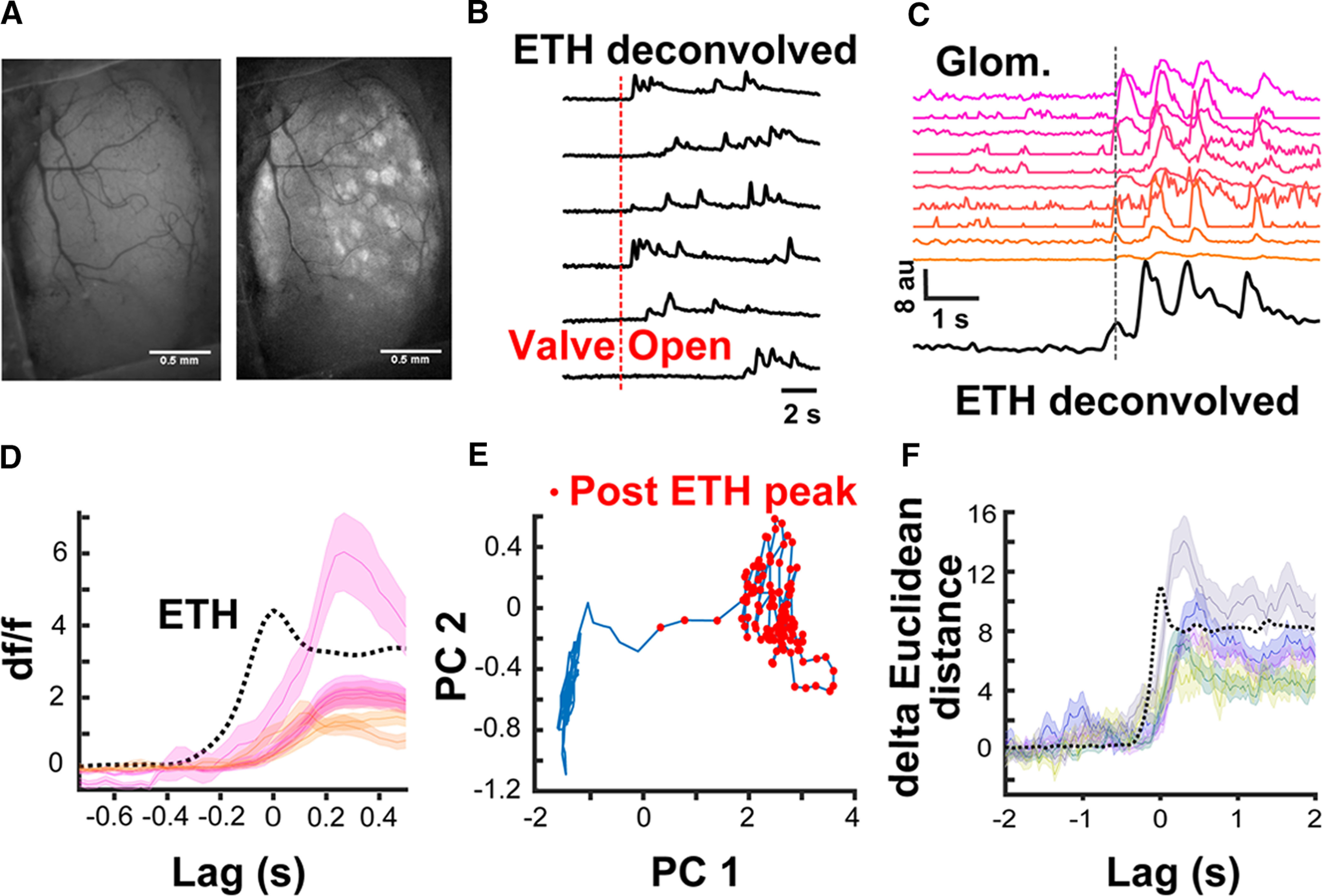
Ethanol plume detected by the ethanol sensor coincide with neural processing in the OB. ***A***, A representative cranial window over the dorsal surface of the OB of a Thy1-GCaMP6f GP5.11 mouse. Left is at rest while right is the SD image after ethanol plume exposure. ***B***, Representative traces showing different times of ethanol plume detection by the sensor after valve opening across six trials. ***C***, Calcium traces from 10 different glomeruli aligned to the first peak of the ethanol plume (dashed line). ***D***, Mean ± SEM traces from six glomeruli averaged over 40 different ethanol plume exposures zoomed in to focus the moment of plume contact. ***E*,** Movement in the first two PCs (accounting 48% of the variance) space post ethanol plume exposure from the data presented in ***C***, ***D***. ***F***, Sum of the Euclidean distance from rest across all the PCs aligned to plume detection from five different imaging sessions. Each trace is the mean ± SEM across 40 ethanol plume presentations during each session.

We asked whether neural processing in the early olfactory pathways is coincident with sensor recordings by conducting widefield calcium imaging over the dorsal OB of head-fixed Thy1GCaMP6f (GP 5.11) mice (Dana et al., 2014). We paired these recordings with ethanol-sensor readings during ethanol plume presentations in a wind tunnel specially designed to create dynamic odor plumes. Figure 6*A* shows resting fluorescence of the dorsal surface of the OB (left) with the SD of the signal after ethanol plume presentation (right). Ethanol plume presentation resulted in mitral and tufted cell activation, which was observed as patterns of activated glomerular networks. Because of the variable time of arrival of the plume from the valve opening (Fig. 6*B*), the glomerular responses within each trial were aligned with respect to the sensor-detected ethanol plume (defined as the first peak in the ETH signal after valve opening). Figure 6*C* shows the difference in fluorescence traces from 10 different glomeruli for a single trial. Figure 6*D* shows the mean activity of six glomeruli from the 10 shown in Figure 6*C*, chosen to span a range of response latencies, to 40 different presentations of the ethanol plume. The mean first peak of the ethanol signal detected by the sensor is also presented. As can be seen, the glomerular activity followed and was locked to the plume encounter detected by the sensor.

We next analyzed how the population-level activity in the OB changed following plume contact by performing principal component analysis (PCA) on the population-level activity in the OB. Figure 6*E* shows the trajectory of the activity in the first two PCs (accounting for 48% of the variance) after plume detection by the alcohol sensor. The trajectory after plume detection (blue line with red dots) moves away from the resting state (blue lines alone). The PCA was then conducted on the glomerular dynamics from individual sessions and the Euclidean distance between all the PCs during the resting and postplume detection was calculated. Figure 6*F* shows that the distance between the PCs increased after plume detection during five individual recording sessions. These results confirm that the plume detection by the alcohol-sensor is coincident with activation of the projection neurons in the OB.

### Behavioral responses to plume contacts in freely behaving mice engaged in odor-guided navigation

**Figure 7.**
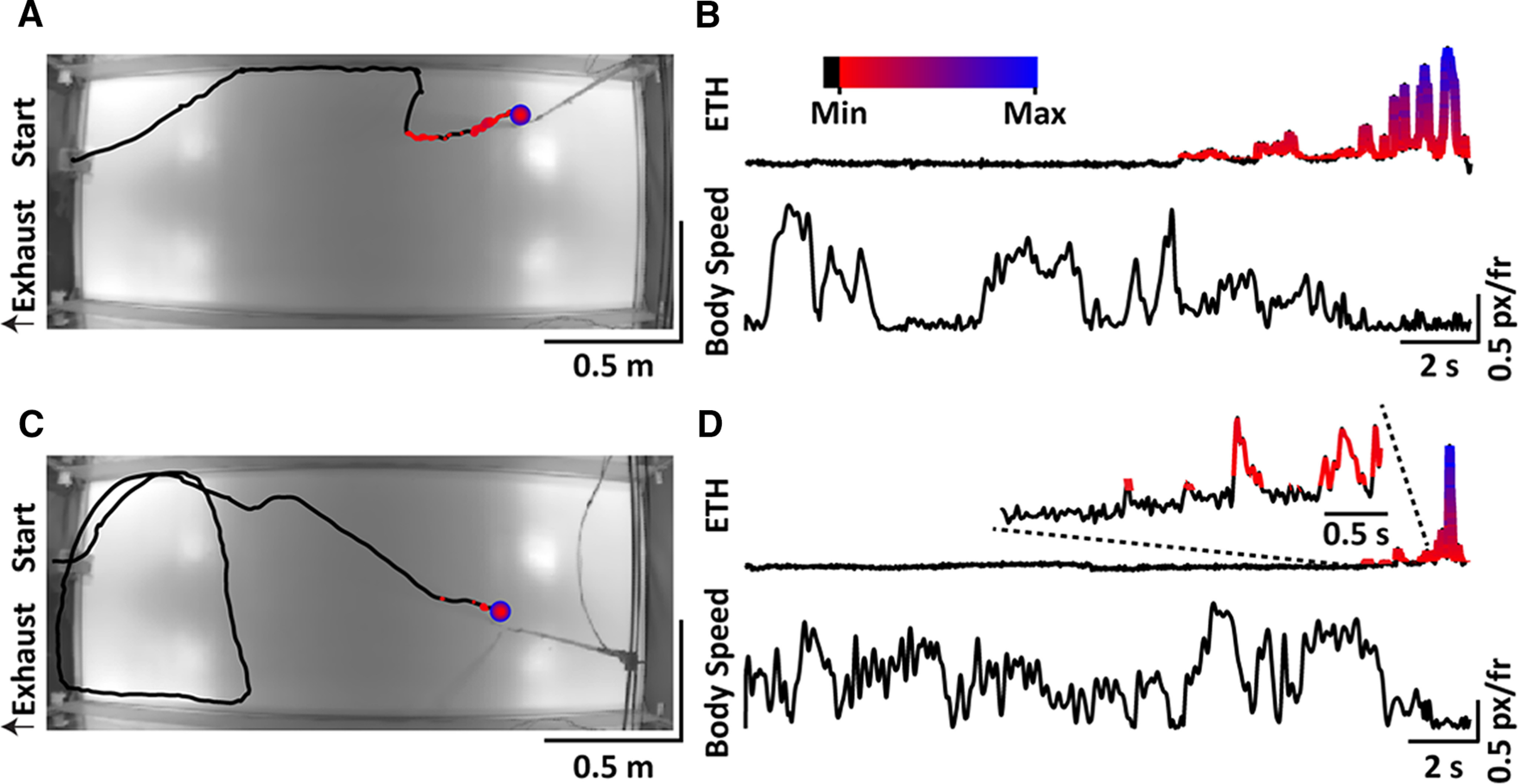
Plume contacts detected by the head-mounted ethanol sensor correlate with behavioral changes in freely behaving rodents. ***A***, An example trajectory of a mouse engaged in odor-guided navigation in our custom-designed arena. Overlaid are the points indicating the locations where plume contacts occurred with the color and size of the points varying by the magnitude of the ethanol plume signal, shown in ***B***, measured by the head-mounted sensor. ***B***, The deconvolved ethanol signal (top), recorded by the head-mounted sensor, with the body speed (bottom) of the animal over the course of a single trial trajectory, shown in ***A***. The plume contacts, set by a threshold, and the magnitude, indicated by the change in color from red (low) to blue (high) are overlaid on the deconvolved ethanol signal. The plume contact is shown to reduce the body speed of the animal. In addition, the plume contact can also be seen to cause a change in the orientation of the animal apparent in ***A***. ***C***, ***D***, Same as ***A***, ***B*** but from another trial where the location of the ethanol source is changed. Inset in the top panel of ***D*** shows the zoomed-in view of the deconvolved ethanol signal within the window indicated by the dashed line.

To study how plume contacts shape behavior in odor-tracking mice, we trained water-deprived mice to associate ethanol with water. Next, they were tasked to find the ethanol source in a large wind tunnel. Each mouse was equipped with head-mounted sensors and its behavior and ethanol plume contact were recorded. Two instances of a mouse engaged in this task are shown in Figure 7. Each mouse readily learned to explore the arena and seek the ethanol source (Fig. 7*A*). The deconvolved ethanol signal was correlated with a decrease in speed in freely behaving mice (Fig. 7*B*,*D*).

**Figure 8.**
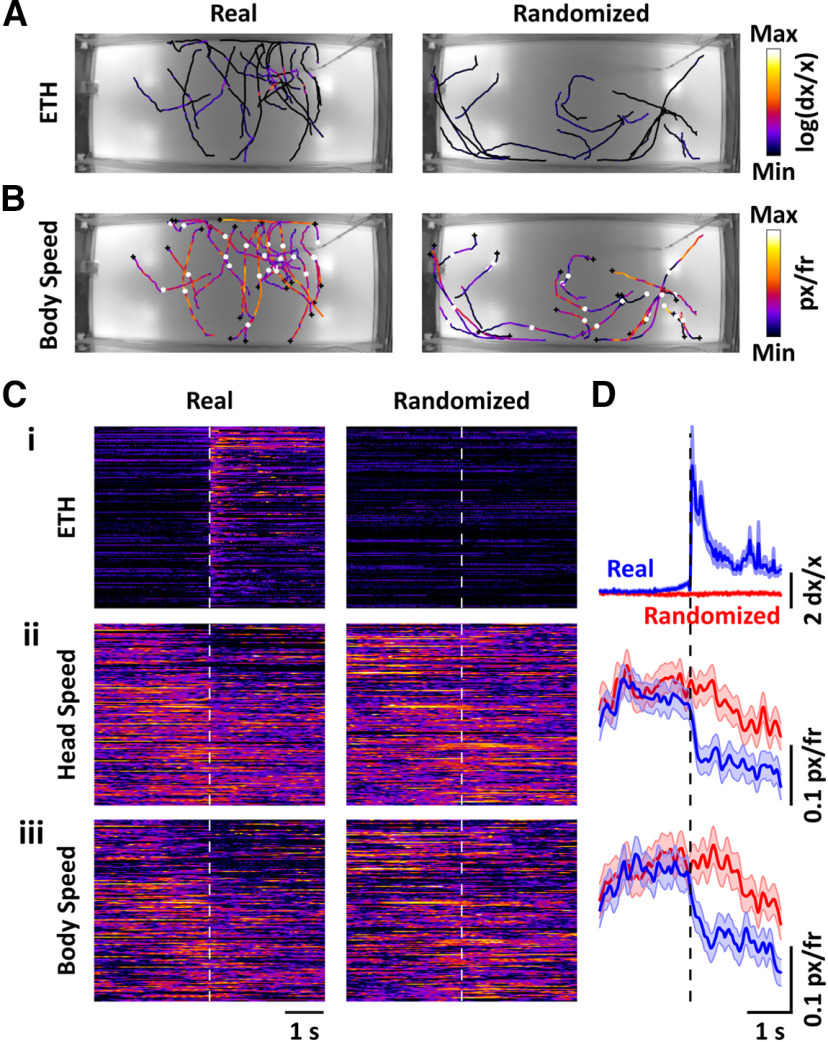
Plume contacts are intermittent and on average are correlated with a reduction in the speed of freely behaving animals. ***A***, Trajectories of multiple animals over different trials during a 2-s period before and after plume contacts for a single ethanol-source location are presented showing that the plume contacts are intermittent and guided by turbulence (left). The color of the trajectories corresponds to the log of the deconvolved ethanol signal (see Materials and Methods). For comparison, equal time-sized trajectories, randomly selected during the non-plume-contact portion of the trials, are presented (right). ***B***, The body speed profile, denoted by the color, peri-plume contacts are presented for the trajectories shown in ***A***. Black plus sign is the beginning of the trajectory while the white circle denotes the position at the time of the threshold crossings signifying plume contacts (left). For the randomized (right) case, the black plus sign again denotes the start of the trajectory while the white circle is the position at the middle of the time window. ***C***, Heatmaps of the log of the deconvolved ethanol signal (***Ci***), head speed (***Cii***), and the body speed (***Ciii***) centered around the time of plume contact (dashed white line) sorted by the distance from the source at the time of contact reveal the decrease in the head and body speeds on plume contacts. For comparison, the randomized heatmaps during the non-plume-contact portion of the trajectories are also presented. Colormap for ***Ci*** corresponds with the colormap in ***A***, while colormaps for ***Cii***, ***Ciii*** correspond with the colormap in ***B***. ***D***, Mean ± SEM from multiple contacts over different trials and animals, shown in ***C***, are presented for the deconvolved ethanol signal (***i***), head speed (***ii***), and the body speed (***iii***), showing clear reduction in the mean head and body speed on plume contact.

We set defined thresholds for each trial from multiple animals over multiple locations, thus binarizing the deconvolved ethanol signal (see Materials and Methods). The trajectories of animals during a window starting 2 s before and ending 2 s after the onset of a contact, defined by the threshold crossing, for a single location are presented in Figure 8*A*. In order to make sure that fluctuations within a single contact were not counted multiple times, we limited the analysis to contacts that were separated by at least 5 s from the previous threshold crossing. Furthermore, the animal had to be >10 pixels away from the source at the time of onset to exclude plume encounters near the source. Plume contact was stochastic with varying spatial patterns during different trials (Fig. 8*A*). In addition, the magnitude of the signal was not spatially predictable. For comparison with random changes in behavior, we randomly selected equal-time sized windows of the trajectories obtained during search without plume contact (defined as sensor reading less than the threshold throughout the duration of the time window). The body speed of the animals decreased on plume contacts, signified by the white circles, as shown in Figure 8*B*. Furthermore, the speed was lower closer to the source as has been previously suggested by statistical analysis of animal movement (Liu et al., 2020).

The ethanol signal (see Materials and Methods), sorted by the distance of the animal from the source at the time of threshold crossing, along with the head and body speed peri-plume contacts (*n* = 177 contacts from 88 trials) are presented in Figure 8*C*. The mean ± standard error of the mean (SEM) of the raw ethanol signal and the head and body speeds before and after plume contacts are presented in Figure 8*D*. Plume contacts, defined by an increase in the ethanol signal, closely aligned with a decrease in body and head speed of the freely behaving animals (Fig. 8*D*). The distribution of the mean head and body speeds 1 s before and after contact was found to be significantly different (*p* = 0.015 for head speed and *p* = 0.0075 for body speed; Kolmogorov–Smirnov test), while no difference in these distributions was observed for the randomized trajectories (*p* = 0.87 for head speed and *p* = 0.785 for body speed). In addition, the mean speed distributions before plume contact were not different from the randomized trajectory means. Hence, body and head speeds significantly decreased on plume contact during odor-guided navigation.

## Discussion

A growing body of literature has focused on understanding the behavioral strategies for odor-source localization in freely moving mammals (Gire et al., 2016; Findley et al., 2020; Gumaste et al., 2020; Liu et al., 2020). However, correlating real-time olfactory information with behavior and physiological recordings from freely moving animals has been a challenging avenue because of the dynamic nature of the olfactory stimulus. Vickers and Baker (1994) first conducted EAG recordings from antennae mounted on freely behaving moths to correlate odor stimuli with behavior. While EAG is an effective measure to monitor odor information, it suffers from degradation of signals over time as well as contamination from changes in air pressure and temperature. Another technology routinely used is the PID (Riffell et al., 2008). However, the PID sensor is bulky and heavy, and thus not feasible for monitoring on freely behaving animals. We therefore used metal oxide gas sensors, specifically alcohol sensors, to monitor real-time olfactory information.

These alcohol sensors are lightweight and inexpensive, and can be easily mounted on the head of freely moving mice. Proximity of the sensor to the nares is critical for making accurate measurements of the signal the animal is sensing. Our paired PID and sensor recordings showed high signal correlation, with nearly identical separation distances as between the sensor and the mouse nares. In addition, fluid dynamics of the ethanol plume in this arena are likely highly correlated across this separation. Furthermore, the results from calcium imaging in OB suggest that the sensor has appropriate sensitivity to detect physiologically relevant fluctuations in ethanol concentrations.

One limitation of using ethanol sensors is their long decay time in response to transient activation. We, therefore, designed kernels resulting from the difference of two exponentials to deconvolve the signal. The deconvolved signals showed a good correlation with the PID responses over time, frequency, and spatial scales. This deconvolution procedure produces similar results as those reported by Martinez et al. (2019). They have also expanded this method to monitor real-time plume detection for mobile robots. We also show good correlation between calcium signals from the mitral and tufted cells in the OB of awake mice and the deconvolved signals from the sensor. In addition, we show that mice reduce both head and body speed in response to odor contacts detected by the sensor. This behavioral change is consistent with that predicted from statistical analyses of mouse behavior during odor-guided navigation (Liu et al., 2020).

There are other limitations to the approach we have taken. The mechanism of detection by the PID is an active process because of a pump that sucks in air, while the metal oxide sensors rely on the adsorption of the analyte to the sensing material, which might account for the shifts we saw in the frequency responses of the ETH signal with respect to the PID signal (Fig. 4), and the difference in the times to reach threshold (Fig. 1*E*). Also, the PID has a larger dynamic range while the oxide sensor’s range is set by the amount of the sensing material, and the adsorption of the analyte. Hence, the saturation of the oxide sensors can occur before the saturation of the PID responses. This could explain the difference in the peak times of the signals generated by the PID and the oxide sensor (Fig. 2*B*), and in the values of the cross-correlation and the PID autocorrelation (Fig. 4). Still, the periodicity of the cross-correlations shows that metal oxide sensors can detect ethanol at the frequency ranges tested. Furthermore, the decrease in the correlations at 15 Hz can be explained by the reduced time in between the odor pulses for the alcohol sensor to clear the analyte in between the pulses.

Direct calibration of the metal oxide sensors requires generating samples of known ethanol concentration and delivering them faithfully to the sensor. This would necessitate specialized equipment, techniques, and facilities not routinely available in a neuroscience laboratory. While this limitation is valid, we show here that the relative signals from the ethanol sensor show good correlation with the relative signals from the PID, allowing us to rely on this lightweight sensor to record real-time olfactory input to freely behaving animals. In the behavioral arena with the turbulent plume, the signals decrease with the distance from the source while the correlation between the signals, on average, remains constant. An explanation for this phenomenon is that both the PID and the ETH signals degrade equally with the distance; hence, the primary variables dictating the correlation are the distance between the PID and the alcohol sensor, and how thin the odor filaments are within the turbulent regime. Furthermore, using an improved dielectric excitation method and the resulting impedance measurements from the improved designs, Potyrailo et al. (2020) have shown linear responses to a varying range of concentrations from metal oxide sensors.

We used alcohol sensors for our study so the mice were trained only on a single odor (ethanol) for the behavioral experiments. While this limits the behavioral repertoire, it had the advantage that the animal was tasked to detect only a single molecule. By following our paradigm for oxide sensors that are sensitive to other compounds, this method could be applied to other odors. A recent publication by Williams and Dewan (2020) has reported detection thresholds of mice for aliphatic alcohols ∼3 orders of magnitude smaller than the sensitivity of the alcohols sensors, which is in the parts per million range, used here. However, under the conditions of turbulent transport (Celani et al., 2014) employed in our study, odor is transported as high concentration packets (whiffs) interspersed with periods of no odor (blanks). Reaching low concentrations on the scale of the thresholds presented by Williams and Dewan (2020) would occur via diffusion, taking place over larger spatial and temporal scales than in our laboratory setting. Another problem that we encountered during experiments with freely behaving animals is tether management within a large, top-sealed wind tunnel. However, the low power consumption of these sensors allowed us to use ultra-thin wires, reducing impact on animal movement.

Despite these limitations, we envision several extensions of this method. In the future, we plan to combine cameras with actuators in a closed-loop system that would follow the animal and move the wires accordingly. This approach is possible using relatively low-cost components (Fee and Leonardo, 2001; Krynitsky et al., 2020). The small footprint of these sensors can be leveraged to allow us to record the olfactory information arriving at the two nares simultaneously. This can allow us to assess whether binaral comparison (Rajan et al., 2006; Khan et al., 2012; Jones and Urban, 2018; Findley et al., 2020) informs behavior during plume tracking in freely behaving animals. Additionally, combining these sensors with other sensors that are specific to other chemicals (Johnston et al., 2012; McGinn et al., 2020) can allow us to study the “foreground” versus “background” problem in the olfactory realm (Riffell et al., 2014).

Our first steps toward measuring real-time olfactory stimuli experienced by a freely behaving rodent will allow detailed quantitative analysis of behavior and provide insights into the behavioral algorithms used to locate an odor source. This technical advance, combined with others for recording or manipulating neural activity in specific brain areas, will help illuminate the brain mechanisms underlying this process that is critical for survival in many species.

## Synthesis

Reviewing Editor: Upinder S. Bhalla, National Centre for Biological Sciences, Tata Institute of Fundamental Research; Decisions are customarily a result of the Reviewing Editor and the peer reviewers coming together and discussing their recommendations until a consensus is reached. When revisions are invited, a fact-based synthesis statement explaining their decision and outlining what is needed to prepare a revision will be listed below. The following reviewer(s) agreed to reveal their identity: NONE.

The reviewers agreed that the authors have addressed their concerns well, and that paper in its current form is nearly ready for publication. They recommend that the title of the paper be modified to make it clear that the method in its current form, and all of the analysis, pertain to alcohols only.

## Author Response

Reviews Response

------------------------------------------------------------------------

We thank the editor and the reviewers for handling this paper. In line with the reviewers’ recommendation “The reviewers agreed that the authors have addressed their concerns well, and that paper in its current form is nearly ready for publication. They recommend that the title of the paper be modified to make it clear that the method in its current form, and the analysis, are applicable to alcohols only.”, we have now updated the title of our manuscript to “Using head-mounted ethanol sensors to monitor olfactory information and determine behavioral changes associated with ethanol-plume contact during mouse odor-guided navigation”.
